# Smokers’ beliefs about the tobacco control potential of “a gene for smoking”: a focus group study

**DOI:** 10.1186/1471-2458-14-1218

**Published:** 2014-11-25

**Authors:** Erika A Waters, Linda Ball, Kimberly Carter, Sarah Gehlert

**Affiliations:** Department of Surgery-Division of Public Health Sciences, Washington University in St. Louis, Saint Louis, MO USA; Department of Social Work, University of Illinois-Edwardsville, Edwardsville, IL USA; George Warren Brown School of Social Work, Washington University in St. Louis, Saint Louis, MO USA; Division of Public Health Sciences, Washington University School of Medicine, Campus Box 8100 660 S. Euclid Ave, Saint Louis, MO 63110 USA

**Keywords:** Gene-environment interaction, Smoking cessation, Tobacco prevention, Health promotion, Personalized medicine, Tobacco control

## Abstract

**Background:**

Several genetic variations associated with nicotine dependence and lung cancer exist. Translating this knowledge into tobacco control policy relies on smokers’ perceptions of the implications of the research. This study explored smokers’ beliefs about the tobacco control uses for research examining genomics, smoking, and addiction.

**Method:**

Smokers (N = 85) participated in one of thirteen focus groups and one interview, stratified by race (eight black, six white) and education (seven < Bachelor’s degree, seven ≥ Bachelor’s degree). Data were analyzed by two independent coders using standard analysis and validation techniques.

**Results:**

Nearly all groups suggested using genetic information for youth-oriented tobacco prevention education. Beliefs about the effectiveness of such actions varied. Many participants believed that providing smokers personalized genetic testing results or informing them about the existence of a gene would not motivate people to quit. All smokers emphasized the need for improved smoking cessation treatment options. Using genomics research to develop gene therapies and personalized drug treatments were also mentioned, yet perceptions of such treatments were mixed. Whereas some participants viewed the possibility positively, others expressed concern about cost and access. Participants who were skeptical of the effectiveness of using genetic information for tobacco control noted that the harms of tobacco use are widely known and genetic information does not add much of a deterrent.

**Conclusion:**

Participants generated several possible tobacco control uses for genomics research findings. Our findings suggest that tobacco control experts should consult with smokers prior to implementing tobacco control measures. The potential public health benefits of genetics and genomics research related to tobacco use cannot be realized until communication strategies that are most likely to encourage and support tobacco avoidance decisions, and minimize mistrust and backlash, are identified.

## Background

Extensive research has examined the association of genetic variants with a variety of tobacco use outcomes, including susceptibility to nicotine addiction, smoking-related diseases, and responsiveness to pharmacological smoking cessation aides [1, 2]. The goal of many of the studies is to use research findings to develop biomedical and behavioral interventions that reduce tobacco morbidity and mortality.

The potential utility of using personal genetic testing to motivate smoking cessation has been debated for several years [3–5]. Early debates have been supplemented by a growing body of research, including meta-analyses and systematic reviews, that suggest that providing smokers with personalized test results for high-risk genetic variants may have limited effectiveness in encouraging cessation [6–8].

Tailoring smoking cessation therapies according to genotype rather than providing general information about smoking risks may be a viable alternative; smokers and physicians report interest in using genetic testing for this purpose, despite several remaining barriers (e.g., perceived unimportance of medications among some smokers, physicians’ concerns about the ability to counsel patients effectively) [9, 10]. However, research examining the efficacy of tailoring smoking cessation therapies by genotype is nascent. To date there is only one published randomized controlled trial that examined the effects of tailoring nicotine replacement therapy (NRT) dose by genotype [11]. It reported that tailoring NRT dose by genotype resulted in higher 6-month abstinence than tailoring dose by phenotype. A follow-up study reported that participants who received more intensive NRT were more successful quitting, but it did not indicate whether NRT dosage or duration mediated the effect of genetic tailoring on cessation [12]. The paucity of data means that several years will pass before adequate efficacy data are published and tailored smoking cessation interventions are widely used. Thus, alternative uses for tobacco-related genomic research should be identified.

Another complication is that people with limited access to healthcare almost certainly will have limited access to genomics technologies. This raises the possibility that translating genomic technologies from bench to bedside might inadvertently worsen existing cancer disparities [13] by virtue of being unavailable to underserved populations. For example, if smokers cannot afford the genotyping needed for individualized cessation therapies, they may be less likely to quit and therefore at increased risk of morbidity and mortality. Thus, the only exposure to genomic information for smokers with limited access to healthcare may be via media outlets and social networks [14].

This paper explores smokers’ beliefs about the potential for genomics research to inform effective tobacco control policies and programs. We solicited smokers’ ideas because the translation of research from basic genomics to policy implementation requires the involvement of key stakeholders [15, 16]. Lack of involvement can impair an intervention’s effectiveness by ignoring critical factors that might otherwise impede implementation. The data presented are a subset of data collected for a study aimed at understanding smokers’ opinions about the discovery of a genetic variation associated with increased risk of severe nicotine addiction and lung cancer [1]. We examined possible differences in beliefs according to race and educational attainment because these variables are strongly related to smoking prevalence and cessation success [17]. This study focused on understanding the dissemination of genomics research findings via mass media rather than through clinics based on the premise that access to clinical genetic testing will likely be limited for many individuals.

## Methods

### Study description

This qualitative study used focus groups to explore participants’ perspectives and beliefs about the link between genetics and nicotine addiction. We chose to use focus groups because we believed that their dynamic and interactive nature would produce higher quality and more complete data than would interviews, due to the relatively low levels of knowledge about genetics in the general population [18]. This is because focus group members tend to stimulate each other’s thinking about topics about which they normally do not converse [19].

Focus groups were stratified by race (African American, White) and educational attainment (<Bachelor’s degree, ≥ Bachelor’s degree), resulting in four strata: higher education/African American (HA), lower education/African American (LA), higher education White (HW), and lower education White (LW). This reflects previous findings in which African American and White participants reported different concerns about the potential misuse of genomic information [20] and reluctance to share that information in racially diverse settings. It is also consistent with surveillance data indicating that the prevalence of tobacco use is lower for people with at least a Bachelor’s degree [17]. Accordingly, each group was facilitated by a moderator and a note-taker who were race-matched to the group.

### Ethical approval and consent

The study team obtained approval to conduct the research from the Washington University School of Medicine Human Research Protection Office and the Siteman Cancer Center Protocol Review Management Committee prior to initiating recruitment. All participants provided signed informed consent upon arrival at the focus group interview site.

### Participants and procedure

Eligibility criteria were: at least 18 years old, having smoked at least 100 cigarettes, currently smoking every or some days of the week, self-identifying as African American or White, not considering oneself to be an expert in genetics, having watched, listened to, or read the news at least once in the past week, being able to demonstrate rudimentary knowledge of the terms “gene” or “genetic”, and being able to speak and read English.

Participants were recruited via flyers distributed in public venues such as community events, and small local businesses such as retail stores, music stores, tattoo parlors, tobacco shops, corner markets, restaurants, and laundromats. Recruitment also occurred via snowball sampling (i.e., word of mouth) and using a registry of research participant volunteers. Participants were screened and scheduled by telephone. Upon arrival at the focus group location, participants were consented and completed a brief survey re-assessing demographic and tobacco history information.

The focus group began with brief introductions and warm-up questions about reasons for smoking and the meaning of “genes” and “genetics”. Terms were then explained briefly to ensure a minimal level of common understanding. Participants shared their thoughts about possible links between genes, diseases, and smoking, and then viewed an Associated Press video clip that described the discovery of a genetic variant associated with nicotine addiction and lung cancer [1]. The video was one minute long and can be viewed at http://youtu.be/sO3X8xBr8YQ. It indicated that three separate scientific teams discovered a genetic variant that conferred an increased risk of severe nicotine addiction and an 80% increased risk of lung cancer. The video also indicated in a very general way that the results give hope for improved smoking cessation treatments. Images of cigarettes, smokers, and technicians pipetting samples were included in the video.

The moderators used a semi-structured interview guide that included broad open-ended questions intended to elicit beliefs about the link between the variant and nicotine addiction and lung cancer (Table 1). Targeted open-ended probes were crafted to assess beliefs about the potential uses of the information and other topics of interest if the topic was not mentioned by any group member spontaneously. Questions and probes were phrased in non-academic, informal language, and approached the target construct indirectly to reduce the risk of biasing responses by asking leading questions [19]. Groups lasted approximately 90 minutes and were audiorecorded. Participants received a $40 gift card and a light meal.

**Table 1:** **Example questions and probes used in focus groups**

	**Pre-video questions and probes**
**1.**	There are lots of reasons why people smoke cigarettes. What is your MAIN reason for smoking?
	• PROBE: “What have you heard about nicotine in relation to cigarettes”
**2.**	What comes to mind when you hear the words “genes” or “genetics”?
**3.**	Sometimes you hear people talking about a genetic risk for a disease. What do you think “genetic risk” means? What does it mean for a person to have “genetic risk”?
	• PROBE: If I had a genetic risk for cancer, does that mean I’d definitely get it?
	• PROBE: What might make one person with a genetic risk more likely to get sick than another person with a genetic risk?
	• PROBE: Thinking about the term “genetic risk,” in your opinion, what does it have to do with smoking?
	**Post-video questions and probes**
**4.**	What do you think of the video?
	• PROBE: Was there something that really made an impression?
	• PROBE: What types of feelings came up as you were watching the clip?
**5.**	When some people hear that genetics and nicotine addiction are related, they do not believe this is true. On the other hand, there are other folks who do believe that genetics and nicotine addiction are linked. What do you believe?
	• PROBE: How important or relevant is this information for you personally?
	• PROBE: How do the concepts presented in the video relate to your personal experiences?
**6.**	If a smoker has this gene, will they definitely not be able to quit?
	• PROBE: What role does willpower have in quitting smoking when someone has a gene for nicotine addiction?
	• PROBE: If you knew you had the gene, would you still try to quit?
**7.**	What could be some of the benefits of people knowing that there’s a genetic basis for nicotine addiction?
	• PROBE: Could you see this information being used to help motivate someone to quit?
	• PROBE: What about being used to discourage people from starting to smoke?
**8**	What could be some disadvantages, or drawbacks of knowing about this information?
	• PROBE: Would YOU want to know whether or not you have the gene?
	• PROBE: How might this information change people’s opinions of smokers?
	• PROBE: How much should people be concerned about discrimination as the result of this news story and stories like it?

Note: Probes only asked if the answers to the main questions did not address topic of interest.

### Data analysis

Audiorecordings were transcribed and examined for accuracy. Data were analyzed using domain analysis [21]. Themes and subthemes were identified deductively, based on the study’s *a priori* areas of interest, and inductively to identify concepts that emerged spontaneously from participants. Two authors (EW, LB) separately listened to and read the transcripts and identified potential themes. Using an iterative process, these themes were discussed and a preliminary codebook was developed. The preliminary codebook was reviewed by SG and feedback was provided. After coding and discussing one transcript from each of the four strata, the codebook was finalized after additional consultation with SG. The remaining transcripts were coded, with the coders conferring regularly to discuss discrepancies. After coding was completed, broad patterns of findings and major themes were identified with special attention given to areas of saturation. NVivo was used for analysis. SG was consulted to provide feedback during each phase of the analysis process and to resolve challenging coding discrepancies.

## Results

### Participant characteristics

We conducted 13 focus groups and one interview that included 85 total participants (see Table 2). There were four groups for each of the HA and LA strata, three groups for the LW strata, and two groups and one interview for the HW strata. Focus group size averaged six participants with a range from three to ten. Due to an error in data collection, the data from the interview (n = 1) was unusable. The findings below are based on the focus group data only (n = 84).

**Table 2:** **Participant characteristics (N = 85)**

	Mean	SD
Age	42.8	12.9
	n	%
Sex		
Men	44	51.8
Women	41	48.2
Race		
African American	52	61.2
White	33	38.8
Education		
<High school	6	7.0
High school/GED	17	20.0
Vo-Tech	5	5.9
Some college	29	34.1
Bachelor’s degree	19	22.4
Postgraduate degree	9	10.6
Smoking frequency		
Daily	78	91.8
Some days	7	8.2
Number of cigarettes smoked daily		
<1	5	5.9
1-9	28	32.9
10-19	27	31.8
20-29	20	23.5
30-39	3	3.5
40+	2	2.4

### Themes

There was no consensus among participants about whether the video presented true information about the relationship between genetics and nicotine addiction and lung cancer (forthcoming manuscript). In every group, at least one participant—and sometimes several participants—made a statement suggesting that he or she was skeptical of the validity of the information. In two of the African American groups, at least one participant had such strong doubts that they had difficulty answering questions about the potential benefits and drawbacks of providing genetic risk information to smokers. Participants were permitted several minutes to discuss their reasons for disbelieving the information. Then, for the purpose of generating conversation and moving the focus groups forward, participants were asked to suspend their disbelief and imagine that the information was true.

Six themes emerged that addressed how research about the smoking-genetics link might be utilized for tobacco control purposes. The themes are described in detail in each of the five subsections below. Importantly, no racial or educational differences emerged about any of the tobacco control themes described below.

#### Improved but potentially costly therapies

Eight of the thirteen (62%) groups mentioned that the discovery of a genetic basis for nicotine addiction and lung cancer might lead to the development of novel treatments or preventive agents. One possibility was to use genetic information to identify the optimal pharmaceutical therapy for a given patient:

[HW] Man 1: “…if they [the researchers] knew more about where that’s [nicotine] affecting their brain, they [the researchers] can…pinpoint [a] more effective drug or treatment perhaps…you can say, ‘Well, we have this drug now to target those people with this gene…’”

Participants also mentioned gene therapy and genetic manipulation:

[LW] Man 2: “…there’s a ton of things that…could be started to prevent…a child from getting [addicted]…even just eliminating it altogether to where something, you know, erases that particular part of genealogy…”

However, the possible cost of such treatments was a concern:

[LA] Woman 1: “…Do you all know if we can alter the gene? Is there something we could do to change it maybe?

Woman 2 responds: “You got to pay for everything.”

As these quotes demonstrate, participants saw the potential for genomics research findings to be used in complex ways to treat or prevent nicotine addiction in adults and children. Yet, statements made by some participants suggest that the cost of such treatments may prohibit access for people without sufficient means.

#### Personalized genetic testing

The potential of personalized genetic testing for smokers arose in all 13 (100%) groups. Participants identified several avenues by which providing genetic test results may or may not affect behavior. However, opinions differed about the extent to which providing test results would motivate people to quit smoking.

[LA] Woman 2: “It would make a difference to me seriously…If I went to the doctor and they said, oh, you got the “double-whammy gene” [quoted from video] for smoking…I’ll be like oh really, I’m going to try harder to quit smoking.”

[HA] Woman 1, in response to moderator question about quitting after genetic testing: “I don’t think so. It has to take something to happen for you to want to…quit.”

In contrast, one participant saw the potential utility of personal genetic testing for motivating smoking cessation, but he was less confident in his ability to put the information into action:

[HW] Man 4: “If I got screened tomorrow and they told me that I had this, I don’t know. I can’t say….I had the threat of losing this tooth, and was able to quit…but once I was healthy, went right back to it.”

Other participants emphasized the idea that providing personalized test results to long-established smokers would be useless unless therapies were available to help them quit:

[LW] Woman 5: “If you come to me tellin’ me this [points to paper summary of video]…I will roll my cigarette in that paper. But if you tell me, ‘Hey, but we got this stuff here. If you drink that, then your chances get 20% better’…then I might consider doing somethin’ with that information.”

These quotes illustrate the complex and multilevel nature of the process by which the provision of personalized genomic testing may or may not influence cessation. The extent to which the information is motivating is highly dependent on the individual. However, the sheer difficulty of quitting raises concerns even in motivated smokers about whether they will be able to transform their intentions into successful actions. Nevertheless, many participants indicated that they would more open to testing if effective treatments were available.

#### Smoking prevention in youth

The potential to use the information to discourage children and adolescents from beginning to smoke was mentioned in 12 of the 13 (92%) groups. However, like personal genetic testing, participants held widely divergent views about its effectiveness:

[LA] Woman 4: “I think [knowing about the gene will make it] harder to start [smoking] because you’re going to get lung cancer.” …

Man 2 responds: “I expect the kids…might get it [be positively affected by the information] before the grownups will.”

Whereas some participants believed that providing young people with their personal genetic test results might be an effective deterrent to smoking, others disagreed by highlighting existing public health messaging surrounding tobacco use:

[HA] Man 3: “They test you early on…and you know that you have it, so that you have a—that with this gene comes a certain death percentage already if you start smoking…they can basically put it in your hands whether you want to start smoking or not.”

Man 1 responds: “We had, ‘it’ll stunt your growth, it’ll give you lung cancer’ and all… it didn’t stop us then. So what’s the difference?”

The possibility that providing information to young people might backfire and increase their susceptibility to experimenting with smoking also arose:

[LW] Man 2: “…I’m not really skeptical, but… what’s the first thing a kid does when you tell him not to do something?”

Some participants were relatively optimistic about the potential of genetic testing to prevent tobacco use in young people, especially in comparison to adults, but others disagreed. Those who were optimistic discussed the idea of young people being able to choose to smoke or not based on having as much information as possible, but others indicated that knowledge did not motivate their smoking behavior. There were also concerns about the information potentially backfiring.

#### Mass media communication

Using the mass media to motivate smokers to quit by disseminating the study findings arose in only four (31%) groups, and then only in response to the moderator’s direct inquiry. Opinions tended to be highly negative:

[LA] Moderator: “If that were on TV right now…”

Man 2 responds: “I think it would be a waste of advertisement.”

[HA] Moderator: “What would the reaction be of…folks if they see this?”

Man 2 responds: I see them turning the TV off. <laughs>

In contrast to these definitive rejections of the information, a few participants believed that responses to mass media-based information would depend on individual situations:

[LW] Woman 3: “I think yes and no… some people will be like, ‘Screw it, I’ve been smokin’ for 42 years, what’s the point?’ And… some people will be like, ‘You know what, my first cigarette was just like a year and a half ago. Let’s do this.’ You know?”

Participants indicated that there would be considerable rejection of mass media-based educational campaigns centered on the study’s findings. This rejection was thought to take both overt actions such as turning the television off, and more cognitively-based rejection. Potential acceptance of the information was voiced infrequently.

#### “We know” smoking is hazardous

Those participants who were skeptical about using genetic information to discourage smoking emphasized that adolescents and smokers already know that smoking is hazardous, yet that knowledge does not affect behavior. These points were raised in ten (77%) of the groups. Some comments focused on school-based tobacco prevention efforts:

[HA] Woman 3: “And it’s not that we don’t know what the consequences of smoking are. You know…when I was in high school…they showed us this movie about smoking, and how your lungs look, and all that…You just say, ‘Okay, I saw that, and…intellectually, I understand.’ But I would choose to smoke.”

Some participants believed that knowledge about the hazardous effects of tobacco was universal, in part due to mass media campaigns. Thus, informing people about the genetic link to nicotine addiction and lung cancer would be superfluous:

[LA] Woman 2: It ain’t going to help nothing because it’s already a known fact that these bad boys are disgusting. It’s not going to help because it’s all over the whole world. Do not smoke…you’re gonna be toe-up [dead]. And they even have commercials and everything.”

Other participants placed their existing knowledge of the hazardous effects in the context of others confronting them about their smoking behavior. This was distressing.

[HW] Woman 1: “Oh my gosh! My daughter came home with [tobacco education material]. That’s a killer, you know?”

Woman 3 responds: “Because we know…We’re gonna die, because we’re smoking. So you don’t have to put it in our face. And the stupid young kids that start smoking, they’re not sitting down watching this stuff.” [angry tone of voice]

The vast majority of participants were well aware that smoking is harmful to health. This awareness came from various sources, including school-based prevention efforts and mass media campaigns that included elements that were highly graphic and generated disgust. Participants were also confronted about the hazards of smoking by loved ones. In that context, relatively emotionally pallid information about a genetic basis for nicotine addiction and lung cancer was insufficiently motivating. Nevertheless, some participants voiced frustration at smoking initiation among young people.

#### Personal agency and motivation

Although opinions diverged widely about the utility of information about the genetic basis for nicotine addiction, the data suggested that, for many participants, the motivational quality of the information was associated with participants’ beliefs about the importance of smoking and smoking cessation as conscious choices. It should be noted that the specific question of the extent to which genetic risk information might be motivating was contingent on participants either believing the information provided or suspending their disbelief. Participants who disbelieved the information cited the importance of personal agency as a reason for their disbelief (data not shown).

Like others, this participant emphasized the ability of the information to help novice smokers decide to quit before addiction becomes too severe:

[HW] Man 3: “If…you had that information up front [before addiction sets in]…you can make it a clear decision…with that information, say “Okay. I’m not—since I’m more susceptible to that, I’m gonna take extra effort not to be caught up in it.”

Other participants focused on the ability of the information to increase willpower when quitting:

[LA] Woman 3: “It [the information] help you say, hey, the gene is not going to control me, I’m going to control it…put my foot down and stomp on it.”

Participants who reported they would be less motivated by the information made statements that were either less focused on personal agency, or made statements that indicated a lack of agency:

[LW] Man 2: “I didn’t really realize it was that—the 80 percent. I mean, both my parents died of cancer and I’m probably right in the top of that number, but you know, as far as quittin’ goes…it just doesn’t even seem like an option…I mean, I, I tried it just doesn’t happen…and I’ve accepted the fact that it’s not gonna happen.”

Although there was near universal agreement that smoking initiation and cessation were conscious choices (data not shown), the role of personal agency in the ability of genetic information to motivate quitting was more varied. Specifically, participants who discussed the motivational value of the information in terms that included agentic statements indicated that the information was more motivating than participants who did not use agentic statements.

## Discussion

Study participants discussed multiple uses for information about the genetic basis for nicotine addiction and lung cancer, despite widespread skepticism about the validity of the research itself. These uses included encouraging smoking cessation and discouraging smoking initiation by providing people with their personal genetic test results, implementing mass media or other health education campaigns, and developing novel biomedical therapeutics.

The perceived effectiveness of the tobacco control strategies varied. Consistent with other research [22], biomedical therapies were seen as potentially highly effective, yet expensive. These concerns were reasonable; adolescent primary care providers reported less willingness to offer testing for genetic variations associated with nicotine addiction if patients had no health insurance [23]. In contrast, beliefs about the potential effectiveness of genomics-based health education were mixed. Some participants thought that providing personalized genomic test results—or even informing people of the discovery of the genetic variant—might encourage some smokers to make a quit attempt and possibly discourage some young people from starting to smoke. Others were more skeptical, explaining that the overwhelming majority of smokers know that smoking harms health and know that they should quit. These findings are consistent with other research, [24–26] although [24] did not report that participants were skeptical about the ability of the information to motivate cessation. Participants who were more open to the potential ability of genetic testing to motivate cessation may have had a stronger focus on personal agency than those who were more skeptical. This is consistent with research indicating that quitting self-efficacy is important for cessation success [27]. Nevertheless, this specific finding needs to be replicated because the relevant focus group data were relatively sparse. Concerns about potential discrimination as a result of testing were also raised in a way similar to prior research (forthcoming manuscript) [22, 26, 28].

Prior studies examined the feasibility and effectiveness of using personalized genetic testing to encourage smoking cessation [10, 22, 24, 25]. We extend this research by examining opinions about a broader array of possible tobacco control measures, including approaches not requiring medical testing (i.e., mass media dissemination). However, formal mass media campaigns may have limited effectiveness, and may not communicate this type of information effectively [28].

Another novel finding is that skepticism about the effectiveness of genetic-based prevention efforts may stem from the belief that, thanks to current tobacco control efforts, people think they already understand smoking risks. In that context, the incremental threat of providing genetic information is insufficient to motivate change. However, although smokers know that smoking is “bad for them” in a general way, they are likely unaware of the extent to which tobacco use harms the entire body [17, 29]. Emphasizing lesser-known consequences of smoking in conjunction with genetic counseling might be beneficial. Paying special attention to appearance-based consequences in campaigns oriented towards younger smokers might increase the relevance for that important subgroup [30]. One reason the information may be insufficiently motivating is because genetic information may be less emotionally impactful than the graphic and visceral images and stories included in many smoking prevention and cessation campaigns, such as the Tips from Former Smokers campaign sponsored by the U.S. Centers for Disease Control (http://www.cdc.gov/tobacco/campaign/tips/).

### Strengths, limitations, and future research

Due to a concerted targeted recruitment effort, most of our study participants (61%) were African American and people with less formal education (68%). This lends confidence that our conclusions are applicable to two demographic groups that experience disproportionate difficulty quitting and morbidity and mortality from smoking-related illnesses [17]. However, we also recruited whites and individuals with more formal education to allow examination of between-group differences that might influence the development of future interventions. Yet we failed to detect between-group differences.

There may be concerns about the demographic and tobacco use characteristics of the study participants. Approximately 50% of people with less than a Bachelor’s degree reported having “some college” experience. This is a higher proportion than in the region from which participants were recruited [31]. It is unlikely that this influenced the study findings, however, because genetics knowledge was also limited in groups with more formal education. We also had exceptional difficulty recruiting smokers with at least a Bachelor’s degree. It is possible that the lower prevalence of smoking among highly educated people is related to stronger negative social norms about smoking, which may have increased reluctance to acknowledge participating in a stigmatized behavior. The smokers in our study smoked an average of 10–19 cigarettes per day. Thus, these results may not generalize to light or intermittent smokers. Future research should examine those vulnerable groups more closely. Future studies might also use quantitative methods to understand the prevalence of these beliefs in different populations, informed by the results of qualitative studies like ours.

## Conclusions

This paper advances the effective translation of basic genomics research into tobacco control interventions, programs, and policies by beginning to understand the perspectives of current cigarette smokers, a key stakeholder group. We found that, for reasons discussed previously, broad mass media-based dissemination associating nicotine addiction and lung cancer with certain genetic variants is unlikely to motivate large numbers of smokers to quit, or to discourage young people from starting. Interpersonal-based dissemination of the information incorporated into school-based tobacco prevention might be effective for some students, but is likely to be met with considerable skepticism and may backfire.

Despite research indicating that personalized genetic testing is ineffective in promoting cessation [6], it might be more effective for a subset of the population. However, this subset needs to be identified and characterized. The effectiveness of genetic testing would likely be maximized if testing is used to target specific biomedical therapies to specific individuals, rather than simply informing patients that they “have the gene.” However, populations with the highest use of tobacco and/or the most difficulty quitting are medically underserved [17]. Unless the genetic testing process and accompanying therapies are inexpensive and easily accessible to medically underserved populations, individuals at highest risk of tobacco-related morbidity and mortality may be those for whom beneficial therapies are inaccessible. This raises the possibility of genetic testing inadvertently exacerbating existing tobacco-related health disparities.

These findings also highlight the fact that genomic risk information does not exist in a vacuum. Rather, it exists in a living context that allows people to reject both the information (see also [32]) and its potential uses. This context is informed by a multitude of factors including, but certainly not limited to, preexisting knowledge about the topic, prior personal experiences, and attitudes about the healthcare system, economics, human development, and in some cases the importance of personal agency for action. Complicating matters further is that these factors are steeped in emotion, as suggested by the statements illustrating the “We Know” theme. Attempting to communicate genomic risk information without attending to at least some of these factors is unlikely to achieve widespread success [33, 34]. This may be especially true for tobacco use, which is highly addictive and has been the subject of health education campaigns for the last 50 years [17].

## References

[1] Amos CI, Wu X, Broderick P, Gorlov IP, Gu J, Eisen T, Dong Q, Zhang Q, Gu X, Vijayakrishnan J, Sullivan K, Matakidou A, Wang Y, Mills G, Doheny K, Tsai YY, Chen WV, Shete S, Spitz MR, Houlston RS (2008). Genome-wide association scan of tag SNPs identifies a susceptibility locus for lung cancer at 15q25.1. Nat Genet.

[2] Russo P, Cesario A, Rutella S, Veronesi G, Spaggiari L, Galetta D, Margaritora S, Granone P, Greenberg DS (2011). Impact of genetic variability in nicotinic acetylcholine receptors on nicotine addiction and smoking cessation treatment. Curr Med Chem.

[3] Bierut LJ, Cubells JF, Iacono WG, Li MD, Madden PA, Nelson EC, Pollock JD, Rutter JL, Swan GE, Vanyukov M (2007). Genetic research and smoking behavior. JAMA.

[4] Carlsten C, Burke W (2006). Potential for genetics to promote public health: genetics research on smoking suggests caution about expectations. JAMA.

[5] McBride CM, Bowen D, Brody LC, Condit CM, Croyle RT, Gwinn M, Khoury MJ, Koehly LM, Korf BR, Marteau TM, McLeroy K, Patrick K, Valente TW (2010). Future health applications of genomics: priorities for communication, behavioral, and social sciences research. Am J Prev Med.

[6] Marteau TM, French DP, Griffin SJ, Prevost AT, Sutton S, Watkinson C, Attwood S, Hollands GJ (2010). Effects of communicating DNA-based disease risk estimates on risk-reducing behaviours. Cochrane Database Syst Rev.

[7] Smerecnik C, Grispen JE, Quaak M (2012). Effectiveness of testing for genetic susceptibility to smoking-related diseases on smoking cessation outcomes: a systematic review and meta-analysis. Tob Control.

[8] Heshka JT, Palleschi C, Howley H, Wilson B, Wells PS (2008). A systematic review of perceived risks, psychological and behavioral impacts of genetic testing. Genet Med.

[9] Shields AE, Najafzadeh M, Schachter AB (2013). Bumps along the translational pathway: anticipating uptake of tailored smoking cessation treatment. Per Med.

[10] Park ER, Kleimann S, Pelan JA, Shields AE (2007). Anticipating clinical integration of genetically tailored tobacco dependence treatment: perspectives of primary care physicians. Nicotine Tob Res.

[11] Marteau TM, Aveyard P, Munafo MR, Prevost AT, Hollands GJ, Armstrong D, Sutton S, Hill C, Johnstone E, Kinmonth AL (2012). Effect on adherence to nicotine replacement therapy of informing smokers their dose is determined by their genotype: a randomised controlled trial. PLoS One.

[12] Hollands GJ, Sutton S, McDermott MS, Marteau TM, Aveyard P (2013). Adherence to and consumption of nicotine replacement therapy and the relationship with abstinence within a smoking cessation trial in primary care. Nicotine Tob Res.

[13] Thomas JC, Irwin DE, Shaugnessy Zuiker E, Millikan RC (2005). Genomics and the public health code of ethics. Am J Public Health.

[14] Pidgeon N, Kasperson RE, Slovic P (2003). The Social Amplification of Risk.

[15] Suther S, Kiros G-E (2009). Barriers to the use of genetic testing: a study of racial and ethnic disparities. Genet Med.

[16] Gehlert S, Colditz GA (2011). Cancer disparities: unmet challenges in the elimination of disparities. Cancer Epidemiol Biomarkers Prev.

[17] U.S. Department of Health and Human Services (2014). The Health Consequences of Smoking—50 Years of Progress. A Report of the Surgeon General.

[18] Christensen KD, Jayaratne TE, Roberts JS, Kardia SL, Petty EM (2010). Understandings of basic genetics in the United States: results from a national survey of black and white men and women. Public Health Genomics.

[19] Ulin PR, Robinson ET, Tolley EE (2005). Qualitative Methods in Public Health: A Field Guide for Applied Research.

[20] Condit CM, Parrott RL, Bates BR, Bevan J, Achter PJ (2004). Exploration of the impact of messages about genes and race on lay attitudes. Clin Genet.

[21] Atkinson S, Abu el Haj M (1996). Domain analysis for qualitative public health data. Health Policy Plan.

[22] Diaz VA, Mainous AG, Gavin JK, Wilson D (2014). Racial differences in attitudes toward personalized medicine. Public Health Genomics.

[23] O’Neill SC, Luta G, Peshkin BN, Abraham A, Walker LR, Tercyak KP (2009). Adolescent medical providers’ willingness to recommend genetic susceptibility testing for nicotine addiction and lung cancer risk to adolescents. J Pediatr Psychol.

[24] Park ER, Kleimann S, Youatt EJ, Lockhart A, Campbell EG, Levy DE, Halbert CH, Schmieder E, Krishna R, Shields AE (2011). Black and white adults’ perspectives on the genetics of nicotine addiction susceptibility. Addict Behav.

[25] O’Neill SC, Lipkus IM, Sanderson SC, Shepperd J, Docherty S, McBride CM (2013). Motivations for genetic testing for lung cancer risk among young smokers. Tob Control.

[26] Docherty SL, McBride CM, Sanderson SC, O'Neill SC, Shepperd JA, Lipkus IM (2011). Young smokers’ views of genetic susceptibility testing for lung cancer risk: minding unintended consequences. J Community Genet.

[27] Gwaltney CJ, Metrik J, Kahler CW, Shiffman S (2009). Self-efficacy and smoking cessation: a meta-analysis. Psychol Addict Behav.

[28] Caburnay CA, Babb P, Kaphingst KA, Roberts J, Rath S (2014). Characteristics of genetics-related news content in black weekly newspapers. Public Health Genomics.

[29] Weinstein ND (1999). What does it mean to understand a risk? Evaluating risk comprehension. J Natl Cancer Inst Monogr.

[30] Williams AL, Grogan S, Clark-Carter D, Buckley E (2013). Appearance-based interventions to reduce ultraviolet exposure and/or increase sun protection intentions and behaviours: a systematic review and meta-analyses. Br J Health Psychol.

[31] **For the Sake of All: A Report on the Health and Well-Being of African Americans in St. Louis and why it Matters for Everyone. Secondary For the Sake of All: A Report on the Health and Well-Being of African Americans in St. Louis and why it Matters for Everyone** [https://forthesakeofall.files.wordpress.com/2014/05/for-the-sake-of-all-report.pdf]

[32] Shepperd JA, Novell CA, O’Neill SC, Docherty SL, Sanderson SC, McBride CM, Lipkus IM (2014). Contemplating genetic feedback regarding lung cancer susceptibility. Ann Behav Med.

[33] Marteau TM, Weinman J (2006). Self-regulation and the behavioural response to DNA risk information: a theoretical analysis and framework for future research. Soc Sci Med.

[34] Hay JL, Meischke HW, Bowen DJ, Mayer J, Shoveller J, Press N, Asgari M, Berwick M, Burke W (2007). Anticipating dissemination of cancer genomics in public health: a theoretical approach to psychosocial and behavioral challenges. Ann Behav Med.

[CR35] The pre-publication history for this paper can be accessed here:http://www.biomedcentral.com/1471-2458/14/1218/prepub

